# Indigenous access to clinical services along the lung cancer treatment pathway: a review of current evidence

**DOI:** 10.1007/s10552-024-01904-1

**Published:** 2024-08-16

**Authors:** Virginia Signal, Moira Smith, Shaun Costello, Anna Davies, Paul Dawkins, Christopher G. C. A. Jackson, Jonathan Koea, Jesse Whitehead, Jason Gurney

**Affiliations:** 1 University of Otago Wellington, Newtown, PO Box 7343, Wellington, 6242 New Zealand; 2 Te Whatu Ora – Southern, Dunedin, New Zealand; 3 Te Whatu Ora – Counties Manukau, Auckland, New Zealand; 4 Department of Medicine, University of Otago, Dunedin, New Zealand; 5 Te Whatu Ora – Waitematā, Auckland, New Zealand; 6 University of Waikato, Hamilton, New Zealand

**Keywords:** Lung cancer, Indigenous, Disparities, Treatment, Survival, Access, Health care, Equity

## Abstract

**Background:**

Lung cancer is a deadly cancer. Early diagnosis and access to timely treatment are essential to maximizing the likelihood of survival. Indigenous peoples experience enduring disparities in lung cancer survival, and disparities in access to and through lung cancer services is one of the important drivers of these disparities. In this manuscript, we aimed to examine the current evidence on disparities in Indigenous access to services along the lung cancer treatment pathway.

**Methods:**

A narrative literature review was conducted for all manuscripts and reports published up until July 20, 2022, using Medline, Scopus, Embase, and Web of Science. Following the identification of eligible literature, full-text versions were scanned for relevance for inclusion in this review, and relevant information was extracted. After scanning 1,459 documents for inclusion, our final review included 36 manuscripts and reports that included information on lung cancer service access for Indigenous peoples relative to non-Indigenous peoples. These documents included data from Aotearoa New Zealand, Australia, Canada, and the USA (including Hawai’i).

**Results:**

Our review found evidence of disparities in access to, and the journey through, lung cancer care for Indigenous peoples. Disparities were most obvious in access to early detection and surgery, with inconsistent evidence regarding other components of the pathway.

**Conclusion:**

These observations are made amid relatively scant data in a global sense, highlighting the need for improved data collection and monitoring of cancer care and outcomes for Indigenous peoples worldwide. Access to early detection and guideline-concordant treatment are essential to addressing enduring disparities in cancer survival experienced by Indigenous peoples globally.

**Supplementary Information:**

The online version contains supplementary material available at 10.1007/s10552-024-01904-1.

## Introduction

Indigenous peoples have higher lung cancer *incidence* and *mortality* than their non-Indigenous counterparts in every population where this has been investigated [1–3]. There have also been significant ethnic and Indigenous differences in lung cancer *survival* noted globally [4–6]: for example, in Aotearoa, Indigenous Māori with lung cancer are 30% less likely to survive their lung cancer once diagnosed than non-Māori [4]. Cancer survival is a marker of access to, and quality of, cancer diagnostics and care within a given country or region [7], because it is plausible to improve survival outcomes by improving access to best-practice care. (In this context, ‘access’ refers to physical access to care.) Survival rates for lung cancer are generally poor, with five-year survival of 10–20% reported in most countries [8, 9].

Prompt referral, early diagnosis, and subsequent timely, best-practice and equitably delivered treatment are critical factors in maximizing cancer survival among those with lung cancer. However, there is growing evidence that this care is not delivered equitably and is likely one factor that drives disparities in lung cancer survival for Indigenous peoples [10, 11].

Addressing these disparities will require us to alter cancer services so that they operate more effectively for Indigenous peoples. However, there remain some key gaps in our understanding: (1) to what extent are there disparities in access to—and the journey through—lung cancer services for Indigenous peoples and (2) to what extent do these disparities impact on survival for Indigenous peoples? In this manuscript, we aimed to examine these questions by reviewing current evidence on Indigenous access to the services along the lung cancer treatment pathway.

## Methods

### Information sources

A search was conducted for all English language manuscripts published up until July 2023 (the date of the review), using the following databases: Ovid Medline; Scopus; Embase; and Web of Science. We also conducted a gray literature search using Google and by searching relevant websites.

### Search

We conducted a narrative literature review using a Boolean approach to literature identification. An example of the search strategy used for one of the scanned databases (Scopus) is shown in Supplementary Material 1.

### Scan for inclusion

The resulting list of manuscripts and reports was initially scanned by VS and MS for inclusion, followed by study abstracts before moving to full-text review; manuscripts were screened out at each of these points. Manuscripts and reports screened out were either not related to indigeneity, not lung cancer specific, or provided data on lung cancer incidence or mortality only, rather than related to treatment, management, or access. The reference lists of manuscripts and reports considered eligible for inclusion were scanned for additional relevant studies, while senior authors (JK and JG) also scanned the final list of included manuscripts to ensure completeness. In addition, manuscripts that were part of our group’s current broader research program in the context of lung cancer, but were in the process of publication, were also included to maximize the completeness and recency of the review.

### Final full-text review

Following the identification of eligible literature, a review of the full-text version of the included manuscripts was completed by one member of the research team (VS). Relevant information from each included manuscript was extracted and collated according to our research questions within a Microsoft Excel spreadsheet (Microsoft Corporation, Washington, U.S.A.). Data extraction criteria included meta-information about the article (author, year, title), study characteristics (design, participants), lung cancer type (e.g., small cell lung cancer), the Indigenous population being studied, and how indigeneity was determined. References were collected and logged in EndNote vX9 (Thomson Reuters, New York, U.S.A.).

## Results

### Selection of studies

**Fig. 1:**
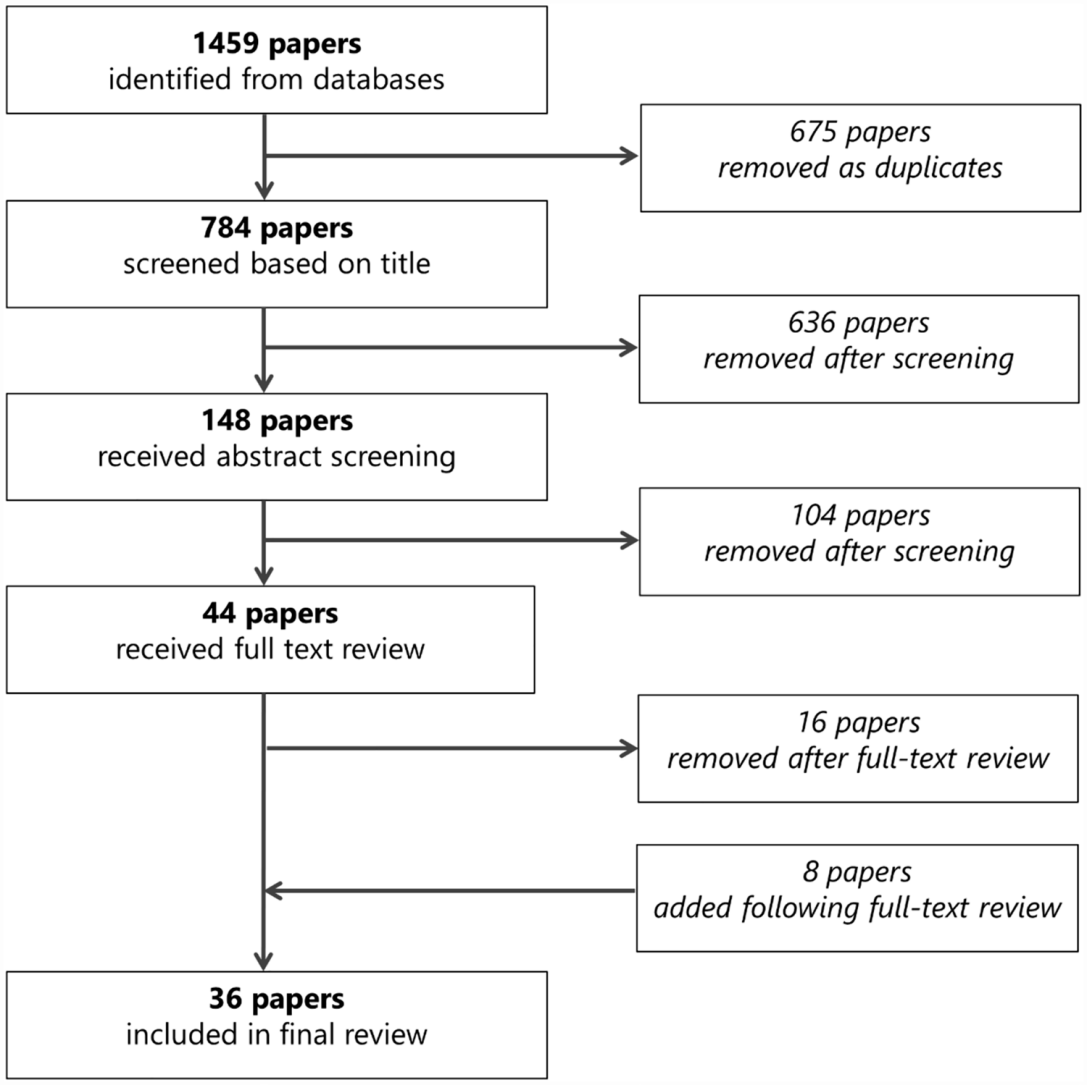
Flowchart for the literature search strategy and results

The flowchart for the literature search strategy is presented in Fig. 1. We reviewed 35 manuscripts and one report [12] that included information on lung cancer service access for Indigenous peoples relative to their non-Indigenous counterparts. A variety of terms were used for Indigenous peoples within the included studies, and we have aimed to use the same terms provided within the given study when reporting their findings (e.g., ‘American Indian’ versus ‘Indigenous American’).

### Characteristics of studies

A table outlining high-level data on all included manuscripts is in Supplementary Material 2. Three studies were qualitative [13–15], while the remainder were quantitative. Included studies were from Aotearoa New Zealand (12) [12–14, 16–24], Australia (8) [11, 15, 25–30], Canada (3) [6, 31, 32], and the USA (10) [33–42], including Hawai’i (3) [43–45]. Of the quantitative studies sample sizes varied, with most studies, including small numbers of Indigenous peoples. Only three studies were based on equal (and matched) cohorts of Indigenous versus non-Indigenous peoples [29, 30, 35]. Additionally, 20 studies stated how ethnicity was ascertained, with six of these studies using self-report [15, 35, 37, 40, 44, 45], which is considered the gold-standard method to determine ethnicity [46, 47]. The remaining studies made no comment on how the Indigenous data had been collected onto the databases.

Papers that reported sociodemographic information all described Indigenous participants as being younger—in Aotearoa New Zealand [18], Australia [25, 27, 29] and the USA [37, 38, 41, 44]—with higher proportions of women (both Australia [27] and the USA [44]), higher burdens of comorbidity (Aotearoa New Zealand [18], Australia [25, 29] and the USA [37]), greater socioeconomic disadvantage (Aotearoa New Zealand [18] and Australia [25, 29]), and being more likely to live more remotely (Australia) [25, 29] than non-Indigenous participants. Stage at diagnosis varied, from being similar across ethnic groups (Australia [25] and the USA [37, 44]) to Indigenous participants being less likely to be diagnosed with localized disease (Australia [29]) and correspondingly more likely to be diagnosed with later-stage disease (Aotearoa New Zealand [18] and the USA [41]).

Our review found evidence of disparities in access to, and the journey through, lung cancer care for Indigenous peoples. Disparities were most obvious in access to early detection and surgery, with inconsistent evidence regarding other components of the pathway. Key findings with respect to Indigenous access to and through the various components of the lung cancer care pathway are detailed in the Discussion section below.

## Discussion

We found that Indigenous peoples have poorer access to early detection of the lung cancer and are less likely to access surgery. We found limited evidence of disparities in access to radiation therapy and systemic therapy and inconsistent evidence regarding likelihood of receiving treatment overall. We found that Indigenous peoples have marginally longer waiting times are less likely to receive guideline-concordant treatment and are less likely to survive their lung cancer than non-Indigenous peoples. We found some evidence that differential access to treatment played a role in survival disparities [29, 39, 41].

### Routes and access to diagnosis

Two papers that used a qualitative design identified many barriers to early diagnosis and treatment of lung cancer for Māori within primary [13] and secondary [14] healthcare in Aotearoa New Zealand. The barriers identified included the quality of the General Practitioner (GP) relationship; GP position in the community; provider communication and its counterpart patient health literacy; issues with access to both primary and secondary care; the need for the health care system to value and respect Māori cultural values; and the need to mitigate the impacts of a cancer diagnosis on the patients’ whānau and facilitate whānau as central enablers in the diagnosis and treatment journeys. An additional qualitative Australian study [15] found some awareness about the causes, and signs and symptoms, of lung cancer among Indigenous community members and health care workers, but a low level of knowledge about lung cancer diagnostic pathways. Several potential service improvements in coordination of care and Indigenous health support services were identified [15].

Two USA-based papers investigated initiatives focused on lung cancer screening. Eligibility for lung cancer screening for American Indians increased from 17 to 21% under the revised United States Preventive Services Task Force screening guidelines, and no evidence of disparities in screening eligibility between American Whites and American Indians were found. This is in comparison to other minority groups, notably African American and Hispanic respondents, who remain less eligible for screening than American Whites under the revised guidelines [40]. The authors concluded that the revised guidelines may perpetuate lung cancer disparities for some ethnic groups. In an HMO (Kaiser Permanente Hawaii) lung cancer screening program using low-dose computed tomography and a Nurse Navigator as a part of clinical care, there were no significantly different screening completion rates by ethnic group. Of the 186 Native Hawaiians who were within the eligibility criteria for the screening study, 149 (80%) completed screening, while 305 (80%) of the 381 non-Hispanic White participants did so [45]. In New Zealand, a recent modeling study found that a national CT lung cancer screening program is likely to be cost-effective and lead to improvements in health and reduction in health inequities for Māori [48]. While some preliminary work has been completed, this screening program is yet to become a reality.

The Te Aho o Te Kahu, Aotearoa New Zealand’s Cancer Control Agency report on Lung Cancer Quality Improvement Monitoring found Māori were more likely to be diagnosed following a presentation to an emergency department than non-Māori (48.9% versus 43.2%) [12]. This finding was supported by another recent study, which showed that 54% of Māori diagnosed with lung cancer had an emergency presentation within 30 days prior to diagnosis, compared to 47% of Europeans [22]. These disparities remained even after adjusting for multiple covariates, including comorbidity and socioeconomic deprivation (adj. OR: Māori 1.21, 95% CI 1.13–1.30). These findings were also consistent with an earlier (2008) study that reported higher proportions of Māori than NZ Europeans entering secondary care following a presentation to an Emergency Department (38% versus 32%) [20].

In terms of access to diagnostic testing, a recent study showed that Māori with lung cancer are similarly likely to have accessed bronchoscopy for the purposes of diagnosis as compared to Europeans with lung cancer [24]. However, when services were significantly disrupted by the COVID-19 pandemic, disparities in access to bronchoscopy were observed in the same context [49]—suggesting that disruptions to usual diagnostics systems may unequally impact Māori.

### Access to surgery

Using nationwide data from 2015 to 2018, Te Aho o Te Kahu found that Māori were less likely to have curative surgery than non-Māori (13.4% versus 17.2%) [12]. One study found that Māori with Stage I and II lung cancer were less likely to receive curative surgery than non-Māori (39.6% versus 49.5%) [16]. Another recent study from Aotearoa New Zealand found that Māori with lung cancer were less likely to have surgery than Europeans (Māori 14%, European 20%; adj. OR 0.82, 95% CI 0.73 to 0.92), particularly curative surgery (Māori, 10%; European 16%; adj. OR 0.72, 95% CI 0.62 to 0.84) [21]. Māori with Stage I and II lung cancers, living within one region of Aotearoa New Zealand, and diagnosed between 2011 and 2018, also appeared to be less likely to have curative surgery than non-Māori (39.6% versus 49.5%, *p* value = 0.027). However, Māori were as likely as non-Māori to have curative treatment overall (70.4% vs 72.5%, *p* value = 0.618) [16].

Three separate Australian studies reported smaller proportions of Indigenous Australians receiving surgery than non-Indigenous Australians. This was reported for: those diagnosed with any lung cancer between 1982 and 2001 (9.5% versus 12.5%) [26]; those diagnosed with non-metastatic NSCLC between 2001 and 2007 (30.8% versus 39.5%) [25]; and those in a single Australian State-based study (11.7% vs 15.9%) [11]. Two studies also reported Indigenous Australians as being 36% [27] or 38% [26] less likely to receive surgery than their non-Indigenous counter parts (adj. odds ratio [OR], 0.64, 95% CI 0.41–0.98 [27] and adj OR, 0.62, 95% CI 0.40–0.96 [26], respectively) [26].

Three studies reported fewer proportions of Indigenous Americans receiving surgery than non-Indigenous. This was reported for those with Stage I NSCLC (58% versus 67%, *p* < 0.0001) [36], and with Stages I–IIIA NSCLC (69% versus 76%, *p* < 0.0001) [41], and in a matched cohort (26.3% versus 40%, *p* = 0.025)) [39]. Also in the USA, American Indian/Alaskan Natives (AI/AN) with non-small cell lung cancer were nearly one-third less likely to undergo surgery than Whites (adj OR, 0.68; 95% CI 0.55–0.83) [38]. By contrast, one study reported no significant differences in proportions of Native Hawaiians receiving surgery than non-Natives (14.4% versus 13.1%) [44], while another reported that AI/AN were more likely to receive surgery than their non-Indigenous counterparts [37]. Finally, Indigenous Americans with Stage I–III NSCLC were 63% less likely to have mediastinal lymph node evaluation (MLNE)—a surgical quality indicator—than Whites [42].

Indigenous Australians were 46% less likely to have surgery for their lung cancer than non-Indigenous when age adjusted only (OR 0.54, 95% CI 0.36–0.80) [25], further inclusion of sex, year of diagnosis, spread of disease, place of residence, comorbidity, and socioeconomic disadvantage explained some of the disparity, with a 30% difference persisting (OR 0.70, 95% CI 0.46–1.05) [25]. Furthermore, non-Indigenous people (who were younger at diagnosis, had fewer comorbidities and more likely to live in major cities) were more likely to have surgical treatment. However, the opposite was true for Indigenous peoples [25]. Another Australian study [11] found the OR between Indigenous and non-Indigenous peoples for receipt of surgery to be 0.46 (95% CI 0.29–0.73, *p* < 0.001, adjusted for age, sex, comorbidity and disease spread). Further adjusting for socioeconomic position and rurality changed the OR to 0.55 (95% CI 0.34–0.87, *p* = 0.01). Further adjusting for having seen a surgeon, possession of private health insurance, and distance to nearest hospital with a cancer-specific multidisciplinary team changed the OR to 0.75 (95% CI 0.04–1.40, *p* = 0.37) [11].

Few papers moved further into explaining why the above disparities in surgical care occurred. A recent Aotearoa New Zealand study that identified lower surgery rates for Māori found that although stage of disease appeared to partially explain this difference, Māori patients remained less likely to receive surgery after adjusting for this and other covariates, including comorbidity [21]. The authors suggest that the remaining disparity is likely to be explained by a combination of a) bias within systems of care that lead to systematic disadvantage (i.e., institutionalized racism), which in turn leads to systematic differences in the availability, affordability, and accessibility of surgery for Māori with lung cancer and b) residual confounding, whereby the study authors inadequately adjusted for important differences between ethnic groups (e.g., incomplete adjustment for stage of disease at diagnosis) [21].

### Access to radiation and systemic therapy

In Aotearoa New Zealand, Māori have been shown to be more likely to be referred to radiation oncology but less likely to be referred to Medical Oncology than New Zealand Europeans [18]. However, later data showed that higher proportions of Māori than New Zealand Europeans received systemic anti-cancer therapy (chemotherapy, targeted therapy, and/or immune therapy) for both NSCLC (32% versus 27%) and SCLC (75.3% versus 70.2%), albeit unadjusted for the confounding impact of factors, including age, stage, and comorbidity [12]. One study found that Māori with lung cancer appeared more likely to access radiation therapy including SABR before adjustment for factors, including differences in age and stage [16]. Once adjusted for these and other factors, Māori appear to receive similar levels of radiation therapy and systemic therapy compared to New Zealand Europeans [16, 21].

In contrast to lower rates of surgery, Indigenous Americans were more likely to undergo radiation therapy than non-Indigenous, either with surgery (11% versus 8%) or alone (2% versus 1%), *p* < 0.0001), with this difference likely at least partially driven by the Indigenous Americans in this study being less likely to have early-stage disease [41]. However, even when focusing on early-stage disease, Indigenous Americans with Stage I NSCLC were more likely to receive radiation therapy than Caucasians (30% versus 19%) [36]. In a USA cohort with a similar stage distribution between 582 AI/AN and 82,696 non-Hispanic whites, authors found that a larger proportion of AI/AN had chemotherapy for their lung cancer than Whites, while a smaller proportion had radiation therapy in this cohort [37]. In Hawai’i, no significant differences in proportions of 229 Native versus 1,165 non-Native Hawaiians receiving either radiation (36% versus 40%) or chemotherapy (6% versus 5%) were reported, although this was based on a relatively small cohort [44].

### Access to precision oncology

Screening for anaplastic lymphoma kinase (ALK) gene rearrangements is an important aspect of management of NSCLC, demonstrated by those who tested positive and treated with tyrosine kinase inhibitors (TKI) in an Aotearoa New Zealand-based study having markedly improved survival rates and times (although not analyzed by ethnicity) [17]. Of the 1,941 participants, 11% of Māori and 12.7% of NZ European were tested for ALK, of the 407 tested for ALK greater prevalence was seen in Māori (6.9% vs 4.4%).

By comparison, a study investigating genetic mutation testing for epidermal growth factor receptor (EGFR) in the USA, found that in AI/AN cases and non-AI/AN controls both diagnosed with adenocarcinoma, about one-third of the participants received EGFR testing with no significant difference in testing between the two groups [35].

There have also been developments within image-guided radiation therapy and intensity-modulated radiation therapy (IMRT) that have improved the ability to deliver high doses of radiation while minimizing dose to adjacent organs. It appeared that Indigenous Americans were similarly as likely to receive IMRT as Whites (Adj OR 1.07, 0.88–1.30); however, Native Hawaiian/Pacific Islanders appeared 20% less likely to receive IMRT than Whites (Adj OR 0.80, 0.45, 1.43) [43].

Access to precision oncology clinical trials is also vitally important as personalized treatments continue to revolutionize cancer care and improve outcomes. In the USA, White participants were consistently overrepresented within trials across four cancer sites (82.3%), including lung cancer trials (85.6%). While Native American/Alaskan Natives were underrepresented across all sites (0.3%) and in lung cancer-specific precision oncology trials (0.2%), the lowest of all ethnic groups studied. In meta-analysis, which weighted individual studies, White participants were overrepresented by 40% in lung cancer trials. In comparison, the numbers for American Indian/Alaskan Native participants were too small to conduct an accurate meta-analysis [34].

### Access to treatment overall

Māori with early-stage (I & II) lung cancer were as likely to have curative treatment overall as non-Māori, in terms of proportions (70.4% vs 72.5%, *p* value = 0.618) (NZ [16]) and odds (adjusted OR 0.80, 95% CI 0.46–1.38) [16]. Data from 2008 suggest that Māori with non-metastatic lung cancer were four times more likely to receive palliative anti-cancer (rather than curative) treatment compared with Europeans (adj OR 4.1, 95% CI 1.4 –12.0, *p* < 0.01) [18]. However, the most recent evidence suggests that Māori receive similar rates of treatment once adjusted for confounding factors, including age and stage of disease [21].

Indigenous Australians were 40% less likely than non-Indigenous Australians to have any treatment for lung cancer [11]. In a matched cohort, also in Australia, fewer Indigenous peoples received chemotherapy, radiotherapy, or surgery than non-Indigenous Australians [29]. Furthermore, after adjusting for histological subtype, stage, and comorbidity Indigenous Australians were over a third less likely than non-Indigenous to receive active treatment for their lung cancer (adj RR 0.65, 95% CI 0.53–0.73) [29].

### Timing of access and guidelines

In Aotearoa New Zealand, there is some evidence that timeliness quality indicators (CQIs) were met less frequently for Māori undergoing surgery for lung cancer although this was based on a small audit [19]. An earlier (2008) Aotearoa New Zealand study found that a large proportion of patients were not managed within internationally recommended timeframes, with longer wait-times for Māori, especially for time from diagnosis to treatment (median 43 days) versus those who were non-Indigenous (29 days, *p* < 0.002) [20]. A recent study found no clear differences between Māori and European patients in the timing of surgery relative to diagnosis, although did find a marginal difference in time to access radiation therapy, with Māori having marginally lower odds of receiving radiation therapy within 0–4-week post-diagnosis (Māori, 27%; European, 32%; adj. OR 0.92, 95% CI 0.82–1.03) and marginally higher odds of receiving this therapy between 4 and 12 weeks of diagnosis (Māori 42%; European 38%; adj. OR 1.11, 95% CI 1.00–1.24) [21].

Indigenous Australians were also less likely to meet guideline-concordant care overall [30], while in the USA, this metric was especially seen for surgical care and post-treatment surveillance received by Indigenous Americans [39]. Indigenous Americans also appeared to have 28% higher odds of substantial treatment delay [33]—defined as more than 10-week post-diagnosis—in the base model (OR 1.28, 95% CI 0.86–1.90), but not in the fully adjusted model (adj OR 1.06, 95% CI 0.70–1.59) [33]. Comparatively, an older study based on data from a single Hawaiian institution found no significant differences in time to primary treatment between Native and non-Native Hawaiians (27.3 versus 28.0 days) [44].

### Survival

Māori have been shown to be less likely to survive at any of one, two, or three years following a lung cancer diagnosis than NZ European/others (37.7% versus 40.9, 21.6% versus 26.4% and 17.5% versus 19.6%, respectively) [12]. While one study has shown that Māori with Stage I and II lung cancer were similarly likely to survive as non-Māori (adjusted OR 1.03, 95% CI 0.53–2.00) [16], another showed that Māori are less likely to survive their lung cancer than non-Māori at each stage of disease. This suggests that Māori have unequal access to curative treatment and emphasizes the importance of updated standards of care and monitoring of care quality for lung cancer [4].

In a 2004 study, Indigenous Australians were nearly 50% less likely to survive their lung cancer than non-Indigenous peoples (unadjusted HR, 1.48; 95% CI 1.14–1.92). Disparities in rates of active treatment accounted for most of the survival disparity, with the difference reducing to 10% following the addition of treatment variables with the hazard ratio, including the null (adj HR 1.10, 95% CI 0.83–1.44). The increased rates of comorbidity among Indigenous peoples accounted for the remaining survival difference between Indigenous and non-Indigenous peoples with lung cancer (adj HR 1.02, 95% CI 0.77–1.35) [29]. In more recent studies, Indigenous Australians remained 32% more likely to die from NSCLC five years after diagnosis (age adj HR, 1.32 95% CI 1.14–1.52); however, the authors did not provide a treatment-related explanation for this disparity [25]. While, Basnayake et al. [28] found that despite receiving similar diagnostic procedures and treatment, Indigenous Australians with lung cancer have poorer 1- and 5-year survival than non-Indigenous Australians.

A study comparing lung cancer survival between First Nations and non-Aboriginals in Canada found that First Nations had 20–25% higher five-year excess mortality than non-Aboriginals [31], while another study also found that First Nations peoples living in Ontario had 20–30% higher lung cancer-specific mortality than non-First Nations peoples [6]. Another smaller study in British Columbia found no clear difference in five-year lung cancer survival between First Nations and non-First Nations peoples [32].

One USA study found no statistically significant differences in survival for Indigenous Americans compared with White patients [37]. By contrast, while Indigenous Americans were more likely to be diagnosed with localized or regional disease [38], AI/ANs were also 9% more likely to die from their lung cancer (adj HR 1.09; 95% CI 1.01–1.19) [38]. Furthermore, although survival improved for both Indigenous Americans and Whites over the study period, Indigenous Americans were found to have shorter survival than Whites [38]. Differential survival was also seen for Native Hawaiians, with death risk 23% higher when compared to non-Native Hawaiians, even after controlling for the independent effects of age, gender and stage (OR, 1.23, 95% CI 1.04–1.45) [44]. Indigenous Americans with Stage I NSCLC were also less likely to survive their cancer, with Indigenous Americans having the poorest survival of all ethnic groups in the study (HR, 1.33; *p* = 0.0194 [Whites as reference group]) [36].

In one study, Indigenous Americans with potentially resectable NSCLC had the worst lung cancer-specific mortality than all other ethnic groups included in the study (*p* < 0.0001) [41]. Indigenous Americans had an estimated 36% higher risk of death than Whites, after adjusting for differences in age, sex, marital status, and histology (adj. HR, 1.36, 95% CI 1.15–1.62). Further controlling for stage at diagnosis, receipt of surgery, and receipt of radiation therapy use partially explained the differences (adj. HR, 1.17; 95% CI 0.98–1.39) [41].

Finally, non-receipt of surgery was associated with significantly poorer (64%) lung cancer-specific survival for First Nations Americans/Alaskan Natives, with survival for those who did not undergo surgery being significantly lower (adj. HR 0.36, 95% CI 0.34–0.37) [39].

### Limitations of available evidence

While the evidence available for this review has highlighted multiple points of inequitable access across the lung cancer pathway for Indigenous peoples, we also noted some weaknesses that prevent us from gaining a full picture of equity in this context. First, the only data available for this review came from either Aotearoa New Zealand, Australia, the USA, or Canada, likely due to the maturity of the collection of data on Indigeneity relative to other countries. This limits the generalizability of our findings to Indigenous populations outside of these regions. Second, even within the included countries, the recording of Indigeneity is not necessarily robust [50] and is often under-counted [51]. This reduces the likelihood that all eligible Indigenous peoples were represented within the data presented in the included studies. Third, on a related matter, the completeness of cancer treatment data varies among jurisdictions, with multiple factors influencing a given study’s accessibility to all relevant treatment information (e.g., availability of all relevant linked health records and completeness of those linked health records). Finally, while many of the included studies have compared access to diagnostics and treatment between Indigenous and non-Indigenous peoples, there are a lack of investigations that have sought to untangle the reasons for any observed differences. For example, while there have been investigations of differences in distance to treatment between Indigenous and non-Indigenous peoples with cancer using Geographic Information Systems (GIS), this remains an under-researched driver of inequities in access to treatment for Indigenous peoples, warranting further investigation.

### Summary of key findings

The following is a summary of the key observations from our review, for each investigated step of the lung cancer care pathway.

- Routes and access to early detection: Indigenous peoples appear more likely to be diagnosed with lung cancer following an acute, emergency presentation, and generally have poorer access to early detection of lung cancer, than non-Indigenous peoples. This likely perpetuates disparities in access to curative treatment further down the pathway. Lung cancer screening may be one solution to these disparities but requires careful planning to ensure such programs are delivered in ways that work best for Indigenous peoples.
- Access to surgery: There is consistent evidence that Indigenous peoples with lung cancer are less likely to receive surgery than non-Indigenous peoples with lung cancer, with this difference only partially explained by factors, such as age, stage of disease, and comorbidity. These findings strongly emphasize the need for consistent monitoring of surgical access and quality performance for Indigenous patients with lung cancer.
- Access to radiation therapy: Based on current evidence, there do not appear to be clear differences in access to radiation therapy between Indigenous and non-Indigenous peoples with comparable lung cancer type and stage. Some studies have shown higher access compared to non-Indigenous peoples, but further research is required to disentangle the extent to which this represents more radical radiotherapy or SABR for early-stage cancer, systematic differences in availability of surgery versus radiation therapy for Indigenous versus non-Indigenous peoples, or a greater receipt of palliative radiation because of differences in stage of disease at presentation.
- Access to treatment overall: There is inconsistent evidence on the likelihood of receiving treatment overall between Indigenous and non-Indigenous peoples with lung cancer. It is likely that the consistent differences in access to surgery should manifest as overall differences in access to treatment, but further data are required to definitively state if this is the case.
- Treatment delays and guidelines: Indigenous peoples appear to have marginally longer waiting times for treatment than non-Indigenous peoples, although the evidence is not substantial. Again, further evidence on the timing of treatment for Indigenous relative to non-Indigenous peoples with lung cancer is required. There is some evidence that Indigenous peoples are less likely to access guideline-concordant treatment, further emphasizing the need for careful monitoring of treatment access and quality for Indigenous patients.
- Survival: There is evidence of survival disparities between Indigenous and non-Indigenous peoples with lung cancer, and the existence of these disparities across stages of disease suggests that unequal access to best-practice treatment is at least a partial driver of these disparities.

### Recommendations

The factors that drive disparities in access to, and the journey through, the lung cancer care pathway for Indigenous peoples are not straightforward to solve. However, we note that the following three recommendations are timely, and deserve further consideration and progression:

- Data collection and monitoring: While we were able to extract and summarize a reasonable volume of information from the available literature, data in the context of Indigenous cancer care and outcomes are relatively sparse. This is due to a combination of factors, including the availability of high-quality ethnicity and treatment data and high-quality linkage between the two. To improve access to, and the journey through, cancer care systems meaningfully for Indigenous peoples, we need to be able to measure and monitor performance in this respect. Conducting this sort of cancer surveillance work for Indigenous peoples is a complex undertaking [50]—but it is achievable and should be prioritized in the context of cancer control for Indigenous peoples.
- Lung cancer screening: Lung cancer screening is one mechanism by which access to an early diagnosis (and thus avoidance of diagnosis following an acute, emergency presentation) can be achieved. In addition, shifting the stage at diagnosis for Indigenous people toward earlier in the disease process would also improve access to curative treatment (particularly surgery). The literature included in our review emphasized the need for screening that is effective for Indigenous peoples; indeed, given strong disparities in incidence, mortality, and survival for Indigenous peoples [1, 3], ensuring that such programs work for these populations is of paramount importance. Each step of a screening program should be carefully designed to ensure that it will maximize access for Indigenous peoples. For example, to maximize uptake of lung cancer screening among Māori, a randomized controlled trial is currently underway in Aotearoa New Zealand examining the best method of inviting people into the screening program [52].
- Increased resourcing of care that works for Indigenous peoples: A key means by which our cancer care pathways can be optimized for our Indigenous patients is to ensure that these pathways are *acceptable* for these patients. Acceptability comes in a number of different forms, from the inclusion of traditional medicines within lung cancer treatment [53], clinical communication in ways that are appropriate for Indigenous peoples [54], to the availability of an Indigenous cancer care workforce that is actively involved in the care of Indigenous lung cancer patients. To help these patients progress on a complex and often-alien cancer care pathway, there is value in investing in Indigenous patient navigators who support and advocate for the Indigenous patient throughout their cancer care [55, 56]. These and similar initiatives aim to make this pathway as familiar as possible for Indigenous patients, with a view to improving access to care and subsequent outcomes.

## Conclusion

This review highlights evidence of disparities in access to, and the journey through, lung cancer care for Indigenous peoples. Disparities were most obvious in access to early detection and surgery, with inconsistent evidence regarding other components of the pathway. These observations are made amid relatively scant data in a global sense, highlighting the need for improved data collection, and monitoring of cancer care and outcomes for Indigenous peoples worldwide. Disparities in lung cancer survival between Indigenous and non-Indigenous peoples, often occurring regardless of stage of disease at diagnosis, emphasize the importance of improvements in access to early detection and guideline-concordant treatment.

## Supplementary Information

Below is the link to the electronic supplementary material.

Supplementary file1 (DOCX 53 KB)

## Data Availability

The data collected for this review are included in the manuscript text and within

## References

[1] Moore SP, Antoni S, Colquhoun A et al (2015) Cancer incidence in indigenous people in Australia, New Zealand, Canada, and the USA: a comparative population-based study. Lancet Oncol 16(15):1483–1492 26476758 10.1016/S1470-2045(15)00232-6

[2] Condon JRBK, Barnes A, Cunningham J (2003) Cancer in Indigenous Australians: a review. Cancer Causes Control 14(2):109–121 12749716 10.1023/a:1023064400004

[3] Gurney J, Robson B, Koea J, Scott N, Stanley J, Sarfati D (2020) The most commonly diagnosed and most common causes of cancer death for Maori New Zealanders. N Z Med J 133:77–96 32994639

[4] Gurney J, Stanley J, McLeod M, Koea J, Jackson C, Sarfati D (2020) Disparities in cancer-specific survival between Māori and Non-Māori New Zealanders, 2007–2016. JCO Global Oncol 6:766–774 10.1200/GO.20.00028 PMC7328125 32511067

[5] Condon JR, Zhang X, Baade P et al (2014) Cancer survival for aboriginal and Torres Strait Islander Australians: a national study of survival rates and excess mortality. Popul Health Metrics 12(1):1 10.1186/1478-7954-12-1 PMC3909914 24479861

[6] Nishri ED, Sheppard AJ, Withrow DR, Marrett LD (2015) Cancer survival among first nations people of Ontario, Canada (1968–2007). Int J Cancer 136(3):639–645 24923728 10.1002/ijc.29024

[7] Seffrin JR (2008) Cancer control as a human right (editorial). Lancet Oncol 9(5):409–411 18452851 10.1016/S1470-2045(08)70113-X

[8] Coleman M, Forman D, Bryant H et al (2011) Cancer survival in Australia, Canada, Denmark, Norway, Sweden, and the UK, 1995–2007 (the International Cancer Benchmarking Partnership): an analysis of population-based cancer registry data. Lancet 377(9760):127–138 21183212 10.1016/S0140-6736(10)62231-3 PMC3018568

[9] Allemani C, Matsuda T, Di Carlo V et al (2018) Global surveillance of trends in cancer survival 2000–14 (CONCORD-3): analysis of individual records for 37 513 025 patients diagnosed with one of 18 cancers from 322 population-based registries in 71 countries. Lancet 391(10125):1023–1075 29395269 10.1016/S0140-6736(17)33326-3 PMC5879496

[10] Stevens W, Stevens G, Kolbe J, Cox B (2008) Ethnic differences in the management of lung cancer in New Zealand. J Thorac Oncol 3(3):237–244 18317065 10.1097/JTO.0b013e3181653d08

[11] Fitzadam S, Lin E, Creighton N, Currow DC (2021) Lung, breast and bowel cancer treatment for Aboriginal people in New South Wales: a population-based cohort study. Intern Med J 51(6):879–890 32638476 10.1111/imj.14967 PMC8362177

[12] Te Aho o Te Kahu. Lung Cancer Quality Improvement Monitoring Report 2021. In: Agency CC, editor. Wellington: Cancer Control Agency; 2021.

[13] Cassim S, Kidd J, Rolleston A et al (2021) Ha Ora: Barriers and enablers to early diagnosis of lung cancer in primary healthcare for Maori communities. Eur J Cancer Care (Engl) 30(2):e13380 33280179 10.1111/ecc.13380

[14] Kidd J, Cassim S, Rolleston A et al (2021) Ha Ora: secondary care barriers and enablers to early diagnosis of lung cancer for Maori communities. BMC Cancer 21(1):121 33541294 10.1186/s12885-021-07862-0 PMC7863263

[15] Page BJ, Bowman RV, Yang IA, Fong KM (2016) A survey of lung cancer in rural and remote Aboriginal and Torres Strait Islander communities in Queensland: health views that impact on early diagnosis and treatment. Intern Med J 46(2):171–176 26550806 10.1111/imj.12948

[16] Lawrenson R, Lao C, Brown L et al (2020) Management of patients with early stage lung cancer—why do some patients not receive treatment with curative intent? BMC Cancer 20(1):109 32041572 10.1186/s12885-020-6580-6 PMC7011272

[17] McKeage MJ, Tin Tin S, Khwaounjoo P et al (2020) Screening for anaplastic lymphoma kinase (ALK) gene rearrangements in non-small-cell lung cancer in New Zealand. Intern Med J 50(6):716–725 31318119 10.1111/imj.14435

[18] Stevens W, Stevens G, Kolbe J, Cox B (2008) Ethnic differences in the management of lung cancer in New Zealand. J Thoracic Oncol: Off Public Int Assoc Study Lung Cancer 3(3):237–244 10.1097/JTO.0b013e3181653d08 18317065

[19] Harrison S, Kim M (2022) Clinical quality indicators of pathways to oncological lung surgery. N Z Med J 135(1556):11–22 35728245

[20] Stevens W, Stevens G, Kolbe J, Cox B (2008) Varied routes of entry into secondary care and delays in the management of lung cancer in New Zealand. Asia Pac J Clin Oncol 4(2):98–106

[21] Gurney J, Davies A, Stanley J et al (2024) Access to and timeliness of lung cancer surgery, radiation therapy, and systemic therapy in New Zealand: a universal health care context. JCO Global Oncol 10:e2300258 10.1200/GO.23.00258 PMC10846779 38301179

[22] Gurney J, Davies A, Stanley J et al (2023) Emergency presentation prior to lung cancer diagnosis: a national-level examination of disparities and survival outcomes. Lung Cancer 179:107174 36958240 10.1016/j.lungcan.2023.03.010

[23] Gurney J, Davies A, Stanley J et al (2024) Equity of travel to access surgery and radiation therapy for lung cancer in New Zealand. Support Care Cancer 32:171 38378932 10.1007/s00520-024-08375-9 PMC10879218

[24] Gurney J, Davies A, Stanley J, et al. Equity of access to pathological diagnosis and bronchoscopy for lung cancer in Aotearoa New Zealand. *New Zealand Medical Journal* 2024; Under review. 10.26635/6965.6422 39509568

[25] Gibberd A, Supramaniam R, Dillon A, Armstrong BK, O’Connell DL (2016) Lung cancer treatment and mortality for Aboriginal people in New South Wales, Australia: results from a population-based record linkage study and medical record audit. BMC Cancer 16:289 27112140 10.1186/s12885-016-2322-1 PMC4845365

[26] Hall SE, Holman CD, Sheiner H (2004) The influence of socio-economic and locational disadvantage on patterns of surgical care for lung cancer in Western Australia 1982–2001. Aust Health Rev 27(2):68–79 15525239 10.1071/ah042720068

[27] Hall SE, Bulsara CE, Bulsara MK et al (2004) Treatment patterns for cancer in Western Australia: does being Indigenous make a difference? Med J Aust 181(4):191–194 15310252 10.5694/j.1326-5377.2004.tb06234.x

[28] Basnayake TL, Valery PC, Carson P, De Ieso PB (2021) Treatment and outcomes for indigenous and non-indigenous lung cancer patients in the Top End of the northern territory. Intern Med J 51(7):1081–1091 32609424 10.1111/imj.14961

[29] Coory MD, Green AC, Stirling J, Valery PC (2008) Survival of indigenous and non-indigenous Queenslanders after a diagnosis of lung cancer: a matched cohort study. Med J Aust 188(10):562–566 18484926 10.5694/j.1326-5377.2008.tb01790.x

[30] Whop LJ, Bernardes CM, Kondalsamy-Chennakesavan S et al (2017) Indigenous Australians with non-small cell lung cancer or cervical cancer receive suboptimal treatment. Asia Pac J Clin Oncol 13(5):e224–e231 26997361 10.1111/ajco.12463

[31] Withrow DR, Pole JD, Nishri ED, Tjepkema M, Marrett LD (2017) Cancer survival disparities between first nation and non-aboriginal adults in Canada: follow-up of the 1991 census mortality cohort. Cancer Epidemiol Biomark Prev 26(1):145–151 10.1158/1055-9965.EPI-16-0706 27965294

[32] McGahan CE, Linn K, Guno P et al (2017) Cancer in first nations people living in British Columbia, Canada: an analysis of incidence and survival from 1993 to 2010. Cancer Causes Control 28(10):1105–1116 28887646 10.1007/s10552-017-0950-7

[33] Adams SV, Bansal A, Burnett-Hartman AN et al (2017) Cancer treatment delays in American Indians and Alaska natives enrolled in medicare. J Health Care Poor Underserv 28(1):350–361 10.1353/hpu.2017.0027 28239006

[34] Aldrighetti CM, Niemierko A, Van Allen E, Willers H, Kamran SC (2021) Racial and ethnic disparities among participants in precision oncology clinical studies. JAMA Netw Open 4(11):e2133205 34748007 10.1001/jamanetworkopen.2021.33205 PMC8576580

[35] Begnaud A, Yang P, Robichaux C et al (2020) Evidence that established lung cancer mortality disparities in American Indians are not due to lung cancer genetic testing and targeted therapy disparities. Clin Lung Cancer 21(3):e164–e168 31759888 10.1016/j.cllc.2019.10.012 PMC7769592

[36] Dalwadi SM, Lewis GD, Bernicker EH, Butler EB, Teh BS, Farach AM (2019) Disparities in the treatment and outcome of stage I non-small-cell lung cancer in the 21st century. Clin Lung Cancer 20(3):194–200 30655194 10.1016/j.cllc.2018.11.004

[37] Emerson MA, Banegas MP, Chawla N et al (2017) Disparities in prostate, lung, breast, and colorectal cancer survival and comorbidity status among urban American Indians and Alaskan Natives. Can Res 77(23):6770–6776 10.1158/0008-5472.CAN-17-0429 PMC5728425 29187399

[38] Fesinmeyer MD, Goulart B, Blough DK, Buchwald D, Ramsey SD (2010) Lung cancer histology, stage, treatment, and survival in American Indians and Alaska Natives and whites. Cancer 116(20):4810–4816 20597131 10.1002/cncr.25410 PMC2950897

[39] Javid SH, Varghese TK, Morris AM et al (2014) Guideline-concordant cancer care and survival among American Indian/Alaskan native patients. Cancer 120(14):2183–2190 24711210 10.1002/cncr.28683 PMC4219619

[40] Narayan AK, Chowdhry DN, Fintelmann FJ, Little BP, Shepard JO, Flores EJ (2021) Racial and ethnic disparities in lung cancer screening eligibility. Radiology 301(3):712–720 34546133 10.1148/radiol.2021204691

[41] Smith CB, Bonomi M, Packer S, Wisnivesky JP (2011) Disparities in lung cancer stage, treatment and survival among American Indians and Alaskan Natives. Lung Cancer 72(2):160–164 20889227 10.1016/j.lungcan.2010.08.015

[42] Tantraworasin A, Taioli E, Liu B, Kaufman AJ, Flores RM (2018) Underperformance of mediastinal lymph node evaluation in resectable non-small cell lung cancer. Ann Thorac Surg 105(3):943–949 29397099 10.1016/j.athoracsur.2017.10.007

[43] Hutten RJ, Weil CR, Gaffney DK et al (2022) Worsening racial disparities in utilization of intensity modulated radiation therapy. Adv Radiat Oncol 7(3):100887 35360509 10.1016/j.adro.2021.100887 PMC8960883

[44] Liu DM, Kwee SA (2004) Demographic, treatment, and survival patterns for native Hawaiians with lung cancer treated at a community medical center from 1995 to 2001. Pac Health Dialog 11(2):139–145 16281691

[45] Oshiro CES, Frankland TB, Mor J et al (2022) Lung cancer screening by race and ethnicity in an integrated health system in Hawaii. JAMA Netw Open 5(1):e2144381 35050353 10.1001/jamanetworkopen.2021.44381 PMC8777569

[46] Cormack D, McLeod M (2010) Improving and maintaining quality in ethnicity data collections in the health and disability sector. Wellington

[47] Jarrín OF, Nyandege AN, Grafova IB, Dong X, Lin H (2020) Validity of race and ethnicity codes in Medicare administrative data compared with gold-standard self-reported race collected during routine home health care visits. Med Care 58(1):e1–e8 31688554 10.1097/MLR.0000000000001216 PMC6904433

[48] McLeod M, Sandiford P, Kvizhinadze G, Bartholomew K, Crengle S (2020) Impact of low-dose CT screening for lung cancer on ethnic health inequities in New Zealand: a cost-effectiveness analysis. BMJ Open 10(9):e037145 32973060 10.1136/bmjopen-2020-037145 PMC7517554

[49] Gurney JK, Dunn A, Liu M et al (2022) The impact of COVID-19 on lung cancer detection, diagnosis and treatment for Māori in Aotearoa New Zealand. N Z Med J 135(1556):23–43 35728246

[50] Sarfati D, Garvey G, Robson B et al (2018) Measuring cancer in indigenous populations. Ann Epidemiol 28(5):335–342 29503062 10.1016/j.annepidem.2018.02.005

[51] McLeod M, Signal V, Gurney J, Sarfati D (2020) Postoperative mortality of indigenous populations compared with non-indigenous populations: a systematic review. JAMA Surg 155(7):636–656 32374369 10.1001/jamasurg.2020.0316

[52] Parker K, Colhoun S, Bartholomew K et al (2023) Invitation methods for Indigenous New Zealand Māori in lung cancer screening: protocol for a pragmatic cluster randomized controlled trial. PLoS ONE 18(8):e0281420 37527237 10.1371/journal.pone.0281420 PMC10393155

[53] Te Aho o Te Kahu (2023) Cancer Control Agency. Rongohia Te Reo, Whatua He Oranga: The Voices of Whānau Māori Affected by Cancer. https://teaho.govt.nz/publications/hui-reports (Accessed 09/05/2023)

[54] Kidd J, Cassim S, Rolleston A et al (2021) Hā Ora: secondary care barriers and enablers to early diagnosis of lung cancer for Māori communities. BMC Cancer. 10.1186/s12885-021-07862-0 33541294 10.1186/s12885-021-07862-0 PMC7863263

[55] Rankin A, Baumann A, Downey B, Valaitis R, Montour A, Mandy P (2022) The role of the indigenous patient navigator: a scoping review. Can J Nurs Res 54(2):199–210 35014886 10.1177/08445621211066765 PMC9109580

[56] Te Aho O Te Kahu (2022) Cancer Control Agency. He Mahere Ratonga Mate Pukupuku - Cancer Service Planning: A vision for cancer treatment in the reformed health system. Wellington, New Zealand: Te Aho o Te Kahu - Cancer Control Agency

