# Supplementary material for: Indigenous access to clinical services along the lung cancer treatment pathway: a review of current evidence
Source: Cancer Causes Control. 2024 Aug 16;35(12):1497–507. doi: 10.1007/s10552-024-01904-1 (PMC11564377; doi:10.1007/s10552-024-01904-1)
Supplement: Supplementary file 1 — Supplementary file1 (DOCX 53 KB) [file 10552_2024_1904_MOESM1_ESM.docx]

## Supplementary Material

**Supplementary Material 1:** Example Boolean search strategy, as used for the Scopus database.

( ( TITLE-ABS ( indigen*  OR  maori*  OR  maaori*  OR  rangatahi  OR  tangata  OR  taangata  OR  tamaiti  OR  tamariki  OR  kirirarau  OR  whanau  OR  "mana whenua"  OR  manawhenua  OR  iwi  OR  aborigin*  OR  "torres straight*"  OR  "first nations"  OR  "first peoples"  OR  ( ( native*  OR  indian*  OR  tribe*  OR  tribal )  W/2  ( america*  OR  alaska*  OR  canad*  OR  hawaii*  OR  australia*  OR  "new zealand*"  OR  austral*  OR  oceani*  OR  pacific* ) )  OR  "pacific island*"  OR  islander*  OR  polynesia*  OR  pacifica  OR  pasefik*  OR  pacifik*  OR  samoa*  OR  "cook island*"  OR  tonga*  OR  niue  OR  niuean  OR  fiji*  OR  tuvalu*  OR  tahiti*  OR  tokelau*  OR  tuamotu  OR  marquesas  OR  "pitcairn island*"  OR  rotuma*  OR  futuna  OR  uvea  OR  "easter island*"  OR  rapanui  OR  "rapa nui"  OR  mangareva  OR  moriori  OR  cherokee  OR  navajo  OR  choctaw  OR  sioux  OR  chippewa  OR  apache  OR  blackfeet  OR  iroquois  OR  inuit*  OR  yupik  OR  eskimo*  OR  anishinaabe  OR  algonquin  OR  nipissing  OR  wyandot  OR  haudenosaunee  OR  cayuga  OR  guyohkohnyo  OR  mohawk  OR  "kanien'kehaka"  OR  oneida  OR  onayotekaono  OR  onondaga  OR  onundagaono  OR  seneca  OR  onondowahgah  OR  tuscarora ) )  OR  KEY ( indigen*  OR  maori*  OR  maaori*  OR  aborigin*  OR  "torres strait*"  OR  "first nations"  OR  "native american*"  OR  "american indian*"  OR  "native hawaiian*"  OR  "alaskan indian*"  OR  "native alaskan*"  OR  "alaskan native*"  OR  "hawaiian native*"  OR  "canadian indian*"  OR  "native canadian*"  OR  inuit*  OR  "pacific island*" ) )

AND

( ( TITLE ( ( ( lung*  OR  bronch*  OR  pulmonary )  W/3  ( cancer*  OR  neoplas*  OR  metasta*  OR  carcinoma*  OR  adenocarcinoma*  OR  sarcoma*  OR  angiosarcoma*  OR  malignan*  OR  blastoma*  OR  hemangioma* ) )  OR  mesothelioma* )  OR  KEY ( ( ( lung*  OR  bronch*  OR  pulmonary )  W/3  ( cancer*  OR  neoplas*  OR  metasta*  OR  carcinoma*  OR  adenocarcinoma*  OR  sarcoma*  OR  angiosarcoma*  OR  malignan*  OR  blastoma*  OR  hemangioma* ) )  OR  mesothelioma* ) ) )

AND

( ( TITLE ( diagnos*  OR  therap*  OR  treatment*  OR  radiation*  OR  radiotherap*  OR  chemo*  OR  service*  OR  surgery  OR  surgical  OR  systemic*  OR  oncolog* )  OR  KEY ( diagnos*  OR  therap*  OR  treatment*  OR  radiation*  OR  radiotherap*  OR  chemo*  OR  service*  OR  surgery  OR  surgical  OR  systemic*  OR  oncolog* ) ) )  AND  ( LIMIT-TO ( LANGUAGE ,  "English" ) )

**Supplementary Material 2:** Characteristics of manuscripts included in this review.

| **Author/Year** | **Paper Title** | **Lung cancer type** | **Indigenous pop** | **Study design** | **Study Location** | **Participants** | **Source of Data** | **Study Period** | **Ascertainment of ethnicity** |
| --- | --- | --- | --- | --- | --- | --- | --- | --- | --- |
| Adams, 2017 | Cancer Treatment Delays in American Indians and Alaska Natives Enrolled in Medicare | n/a | AI/AN | Retrospective cohort | USA | Medicare enrolees identified as NHW or AI/AN race, 65 years and older, diagnosed with first primary invasive lung cancer, not diagnosed at death | Medicare enrolment files and Indian Health Service Care System records linked to SEER cancer registry data | 2001 - 2007 | Not noted - except as identified on the database/s |
| Aldrighetti et al, 2021 | Racial and Ethnic Disparities Among Participants in Precision Oncology Clinical Studies | n/a | AI/AN | Cross-sectional | USA | US-based cancer studies incorporating precision oncology objectives for reporting of ethnicity. 93 studies encompassing 5867 enrolees across four cancer sites | Clinicaltrials.gov registry database | December 2020 - April 2021 | Not noted |
| Basnayake et al, 2021 | Treatment and outcomes for indigenous and non-indigenous lung cancer patients in the Top End of the Northern Territory |  | Indigenous Australians | Retrospective cohort | Top End of the Northern Territory, Australia | 317 adults newly diagnosed with Lung cancer (91 indigenous (28.7%) and 226 (71.3%) non-indigenous) | Clinical note review at Royal Darwin Hospital, and general practitioner and other hospital networks | 1 Jan 2010 - 31 Dec 2014 | Verified the information about indigenous status by medical note review |
| Begnaud et al, 2020 | Evidence That Established Lung Cancer Mortality Disparities in American Indians Are Not Due to Lung Cancer Genetic Testing and Targeted Therapy Disparities | All lung cancers but majority were NSCLC - adenocarcinoma | AI/AN | Retrospective case control | Minnesota, USA | 200 cases and 164 matched controls, matched on histology, smoking status, sex, and age. | Clinical Note Review | 2010 - ? | Self-identified as AI/AN in the electronic health record |
| Cassim et al, 2021 | Ha Ora: Barriers and enablers to early diagnosis of lung cancer in primary healthcare for Māori communities | n/a | NZ Māori | Kaupapa Māori qualitative | 1 region of NZ, five rural localities in the Midland region | Adults with cancer, whanau and community members. Staff at the local primary healthcare centre. | 9 community hui and 9 primary healthcare provider hui | Not noted | Not noted |
| Coory et al, 2008 | Survival of Indigenous and non-Indigenous Queenslanders after a diagnosis of lung cancer: a matched cohort study | All lung cancers | Indigenous Australians | Retrospective cohort | Queensland, Australia | 158 Indigenous and 152 non-Indigenous patients (frequency-matched on age, sex and rurality) diagnosed with lung cancer and treated in Queensland public hospitals | Clinical note review | 1996 - 2002 | Indigenous status as routinely recorded in Queensland public hospitals' patient administration systems |
| Dalwadi et al, 2019 | Disparities in the Treatment and Outcome of Stage I Non-Small-Cell Lung Cancer in the 21st Century | [Stage I  NSCLC](https://www-sciencedirect-com.wmezproxy.wnmeds.ac.nz/topics/medicine-and-dentistry/stage-1) | AI - American Indian | Retrospective cohort | USA | 62,312 Stage 1 -NSCLC patients age 60+ years, 198 AI (O.3%) | SEER (Surveillance, Epidemiology, and End Results) database | 2004 - 2012 | Not noted |
| Emerson, 2017 | Disparities in Prostate, Lung, Breast, and Colorectal Cancer Survival and Comorbidity Status among Urban American Indians and Alaskan Natives | Nil noted | AI/AN | retrospective cohort | Northern California, USA | 83,278 (122 AI/AN with lung cancer and 16,933 NHW) enrollees of Kaiser Permanente diagnosed with primary invasive breast, prostate, lung, or colorectal cancer | Tumour registry and clinical note review | 1997 - 2015 | Ethnicity obtained from Kaiser Permanente demographic files, which includes data primarily obtained through patient self-report during outpatient visits or hospitalizations. |
| Fesinmeyer et al, 2010 | Lung cancer histology, stage, treatment, and survival in American Indians and Alaska Natives and whites | Non-small cell carcinoma | USA - Native American/Alaskan Native | Retrospective cohort - with comparative study time periods | USA | 198,946 participants diagnosed with non-small cell lung cancer - NA/AN n= 1050 (0.53%) versus Whites n=197,896 (99.47%) | Surveillance, Epidemiology, and End Results (SEER) program | 1973 - 2006 (1973-1999 and 2000-2006) | Not noted |
| Fitzadam et al, 2021 | Lung, breast and bowel cancer treatment for Aboriginal people in New South Wales: a population-based cohort study |  | Indigenous Australians | Retrospective cohort | New South Wales, Australia | 230 Indigenous and 8597 non-Indigenous adults aged over 17 diagnosed with a first primary lung cancer | NSW Cancer Registry linked to - Admitted Patient Data Collection, Medicare Benefits Schedule, Pharmaceutical Benefits Scheme and Outpatient Radiation Oncology Dataset | Jan 2009 - June 2012 | Aboriginal and Torres Strait Islander status was assigned using information in the NSWCR, APDC, NSW Emergency Department Data Collection and the Cause of Death Unit Record File using a ‘weight of evidence’ method |
| Gibberd el al, 2016 | Lung cancer treatment and mortality for Aboriginal people in New South Wales, Australia: results from a population-based record linkage study and medical record audit | Non-small cell carcinoma/ non-metastatic NSCLC for treatment analyses | Indigenous Australians | Retrospective cohort | New South Wales, Australia | 20,154 people 18+ years diagnosed with primary non-small cell lung cancer - Aboriginal n= 341 (1.7%) versus non-Aboriginal n= 19,813 | NSW central cancer registry records linked to hospital inpatient episodes and deaths | 2001-2007 | A person was determined to be Aboriginal if they were listed as Aboriginal and/or Torres Strait Islander in any of their linked records |
| Gurney et al, 2023 | Emergency presentation prior to lung cancer diagnosis: A national-level examination of disparities and survival outcomes | All lung cancers | Māori | Retrospective cohort | Aotearoa New Zealand | 27,869 participants (5,601 Māori, 1,267 Pacific, 1,180 Asian, 123 MELAA/Other, 19,698 European) | New Zealand Cancer Registry - linked to public and reporting private hospital inpatient records, emergency department and outpatient records, the mortality collection, and Primary Health enrolment data | 2007-2019 | Prioritised ethnicity derived from the NZCR |
| Gurney et al, 2023 | Equity of travel to access surgery and radiation therapy for lung cancer in New Zealand | All lung cancers | Māori | Retrospective cohort, using GIS analysis to determine the distance between the location where a patient lived at the time of their procedure, and the location where their procedure occurred | Aotearoa New Zealand | 27,869 participants (5,601 Māori, 1,267 Pacific, 1,180 Asian, 123 MELAA/Other, 19,698 European) | New Zealand Cancer Registry - linked to public and reporting private hospital inpatient records, emergency department and outpatient records, Primary Health enrolment data, and the mortality collection | 2007-2019 | Prioritised ethnicity derived from the NZCR |
| Gurney et al, 2024 | Access to and timeliness of lung cancer surgery, radiation therapy, and systemic therapy in New Zealand: A universal health care context | All lung cancers | Māori | Retrospective cohort | Aotearoa New Zealand | 27,869 participants (5,601 Māori, 1,267 Pacific, 1,180 Asian, 123 MELAA/Other, 19,698 European) | New Zealand Cancer Registry, a population-based registry of all invasive cancers, except nonmelanoma skin cancer, diagnosed in New Zealand | 2007-2019 | Prioritised ethnicity derived from the NZCR |
| Gurney et al, under review | Equity of access to pathological diagnosis and bronchoscopy for lung cancer in Aotearoa New Zealand | All lung cancers | Māori | Retrospective cohort, using GIS analysis to determine the distance between the location where a patient lived at the time of their diagnostic procedure, and the location where their procedure occurred | Aotearoa New Zealand | 27,869 participants (5,601 Māori, 1,267 Pacific, 1,180 Asian, 123 MELAA/Other, 19,698 European) | New Zealand Cancer Registry, linked to public and reporting private hospital inpatient records, emergency department and outpatient records, Primary Health enrolment data, and the mortality collection | 2007-2019 | Prioritised ethnicity derived from the NZCR |
| Hall et al, 2004 | Treatment patterns for cancer in Western Australia: does being Indigenous make a difference? | All lung cancers | Indigenous Australians | Retrospective cohort | Western Australian (WA) | 13,377 participants (Indigenous= 274 (2.0%) versus non-Indigenous n=13103 (98.0%)) who had a lung cancer registration in the state-based WA Record Linkage Project | WA Record Linkage Project - routine administrative data set | 1982-2001 | Due to variability in the recording of Indigenous status, any mention in any hospital, cancer or death record was used in this study |
| Hall et al, 2004 | The influence of socio-economic and locational disadvantage on patterns of surgical care for lung cancer in Western Australia 1982-2001 | All lung cancers | Indigenous Australians | Retrospective cohort | Western Australian (WA) | 12708 participants (Indigenous n= 269 (2.1%) versus non-Indigenous n=12439 (97.9%)) who had a lung cancer registration in the state-based WA Record Linkage Project | WA Record Linkage Project - routine administrative data set | 1982-2001 | Not noted |
| Harrison and Kim, 2022 | Clinical quality indicators of pathways to oncological lung surgery | n/a | NZ Māori | Retrospective cohort | Dunedin Public Hospital (serves 1 region of Aotearoa New Zealand - Southern Health District) | 108 patients who underwent curative intent oncological lung surgery and were discussed at a lung cancer MDT meeting. 91 New Zealand European (84.3%); 11 Māori (10.2%); 3 European (2.8%); 1 Samoan (0.9%); 1 Asian (0.9%); and 1 Middle Eastern (0.9%). | Clinical note review | 2014-2020 | Not noted |
| Hutten et al, 2022 | Worsening Racial Disparities in Utilization of Intensity Modulated Radiation Therapy | n/a | USE - Native American/ Eskimo | Retrospective cohort | USA | 1,010,292 participants who received radiation therapy as part of definitive treatment, with 485,340 (48.0%) receiving IMRT. Lung cancer 210,222 (76.1) received CRT and 66,192 (23.9) IMRT. | National Cancer Database | 2004 - 2017 | Not noted |
| Javid et al, 2014 | Guideline-concordant cancer care and survival among American Indian/Alaskan Native patients |  | AI/AN | Retrospective cohort | USA | 338,204 patients (211 AI/AN with lung cancer and 64,240 White) who were diagnosed at age >/=65 years with breast, colon, lung, or prostate cancer | SEER (Surveillance, Epidemiology, and End Results) database, linked to Medicare enrollment and claims data | 1996 - 2005 | Individuals were classified as AI/AN if they had a reservation residence, evidence of medical coverage through the IHS, or their medical record indicated AI/AN ethnicity. |
| Kidd et al, 2021 | Ha Ora: secondary care barriers and enablers to early diagnosis of lung cancer for Māori communities | n/a | NZ Māori | Kaupapa Māori qualitative | 1 region of NZ, five rural localities in the Midland region | Adults with cancer, whanau (families), and other community members. Staff at the local primary healthcare centre, including General Practitioners and nurses. | 9 community hui and 9 primary healthcare provider hui | Not noted | Not noted |
| Lawrenson et al, 2020 | Management of patients with early stage lung cancer - why do some patients not receive treatment with curative intent? | All lung ca - Stage I and II | NZ Māori | Retrospective cohort | Midland Cancer Network region of Aotearoa New Zealand | 583 patients diagnosed with early stage (I or II) lung cancer (ICD code: C33, C34), 169 Māori and 414 non-Māori | Midland Lung Cancer Register (data collected at MDMs within the region and the NZCR), for missing data and those with no record of treatment patient notes were searched to ascertain the reasons for no treatment | 2011-2018 | Not noted |
| Liu and Kwee, 2004 | Demographic, treatment, and survival patterns for Native Hawaiians with lung cancer treated at a community medical center from 1995 to 2001 | NSCLC | Native Hawaiians | Retrospective cohort | Single institution - Hawaii, USA | 1,394 participants with NSCLC, of those, 229 patients self-reported Native Hawaiian ancestry. | Queen’s Medical Center (QMC) Oncology Data Registry, part of the SEER database | 1995 - 2001 | Self-report |
| McKeage et al, 2020 | Screening for anaplastic lymphoma kinase (ALK) gene rearrangements in non-small-cell lung cancer in New Zealand | NSCLC | NZ Māori | Prospective cohort observational study | Northern region of NZ | 3130 patients with tracheal, bronchial or lung cancer with morphology codes indicating non-squamous or not otherwise specified morphological subtypes. Of these, 407 patients who were tested for ALK. | New Zealand Cancer Registry | Jan 2010 - July 2016 | Not noted |
| McGahan et al, 2017 | Cancer in First Nations people living in British Columbia, Canada: an analysis of incidence and survival from 1993 to 2010 | All lung cancers | First Nations | Retrospective cohort study | British Columbia, Canada | 44,595 lung cancers of which 394 occurred among First Nations peoples | Probabilistic linkage using the British Columbia Cancer Registry (BCCR), Statistics Canada SubProvincial Population Projections 2014 and the 2014 FNHA First Nations Client File | 1993-2010 | INAC Indian Registry |
| Narayan et al, 2021 | Racial and Ethnic Disparities in Lung Cancer Screening Eligibility | n/a | American Indian | Retrospective analysis of cross-sectional survey data | 20 states of USA | Survey respondents without a history of lung cancer aged 55-79 years (ie, under the previous guidelines) or aged 50-79 years (ie, under the revised guidelines) were included | Behavioral Risk Factor Surveillance System survey | 2019 | Self-reported |
| Nishri et al, 2020 | Cancer survival among First Nations people of Ontario, Canada(1968–2007) | All lung cancers | First Nations | Retrospective cohort study | Ontario region of Canada | 140,000 Ontario First Nations people | Probabilistic linkage to Ontario Cancer Registry (OCR) | 1968-2007 | Not noted |
| Oshiro et al, 2022 | Lung Cancer Screening by Race and Ethnicity in an Integrated Health System in Hawaii | n/a | Native Hawaiian | Retrospective cohort | Hawaii - USA | 1030 eligible LCS program members from a Health Maintenance Organization (Kaiser Permanente Hawaii), Native Hawaiian n=186 participants [18.1%] | Clinical note review - Electronic medical records | January 1, 2015 - December 31, 2019 | Self-reported race and ethnicity information available in the Electronic medical records |
| Page et al, 2016 | A survey of lung cancer in rural and remote Aboriginal and Torres Strait Islander communities in Queensland: health views that impact on early diagnosis and treatment | n/a | Aboriginal and Torres Strait Islanders | Survey | Queensland, Australia | 2 patients referred with symptoms suspicious of lung cancer or confirmed lung cancer (one rural/remote and one urban); 14 Indigenous health workers; 51 community members aged 18 years and over. | Qualitative interviews and demographic survey | 2013 | Self-report survey question |
| Smith et al, 2011 | Disparities in lung cancer stage, treatment and survival among American Indians and Alaskan Natives | Potentially resectable (stages I-IIIA) non-small cell lung cancer | USA - American Indians and Alaskan Natives | Retrospective cohort | USA | 69,138 people diagnosed with Stage I-III NSCLC - 274 (0.4%) were AI/AN, 56,029 (81.0%) were white, 6251 (9.0%) were black, 2615 (4.0%) were Hispanic, and 3969 (5.6%) of other ethnicity | SEER database | 1988 - 2006 | Ethnicity was coded in accordance with the federal Office of Management and Budget Directive |
| Stevens et al, 2008 | Varied routes of entry into secondary care and delays in the management of lung cancer in New Zealand | All lung cancer | NZ Māori | Retrospective cohort | Auckland and Northland regions, Aotearoa New Zealand | 565 participants diagnosed with primary lung cancer and had some component of initial management in secondary care. Māori n=93 | Clinical note review - Electronic and paper medical notes | 2004 | Ethnicity was obtained from the hospital registration form and compared with that recorded in the national NZ Cancer Registry |
| Stevens et al, 2008 | Ethnic differences in the management of lung cancer in New Zealand | All lung cancer | NZ Māori | Retrospective cohort | Auckland and Northland regions, Aotearoa New Zealand | 565 participants diagnosed with Lung Cancer in 2004. Comprised of: European 378 (67%), Maori 95 (17%), Pacific Peoples 56 (10%), Asian 23 (4%), and other or unknown ethnicity 13 (2% | Clinical Note Review - Electronic and paper medical notes | 2004 | Ethnicity was obtained from the hospital registration form and was compared with that recorded in the national NZ Cancer Registry |
| Tantraworasin et al, 2018 | Underperformance of Mediastinal Lymph Node Evaluation (MLNE) in Resectable Non-Small Cell Lung Cancer | NSCLC Stage I - IIIa | USA - Native American/Alaskan Native | Retrospective cohort | USA | 86,721 adults diagnosed with non-small cell lung cancer stage I to IIIA - 73,034 (84.2%) with MLNE and 13,687 (15.8%) without. | SEER - Surveillance, Epidemiology, and End Results Program database | 2004 - 2013 | Not noted |
| Te Aho o te Kahu, 202 | Lung Cancer Quality Improvement Monitoring Report 2021 |  | NZ Māori | Retrospective cohort | Nationwide, NZ | 8,577 participants with a primary diagnosis of lung cancer (1,855 Māori and 5,828 NZ European/other) | National Cancer Registry (NZCR) linked to routinely available national administrative data sources (NMDS, Pharms, Radiation Dataset and NNPAC) | 1 January 2015 to 31 December 2018 | Not stated |
| Withrow et al, 2017 | Cancer Survival Disparities Between First Nation and Non-Aboriginal Adults in Canada: Follow-up of the 1991 Census Mortality Cohort | All lung cancers | First Nations | Retrospective cohort | Canada | 2 million respondents to the 1991 Canadian Long Form Census | Probabilistic linkage from Census to cancer and death registries | 1991-2009 | 1991-2009 |
| Whop et al, 2017 | Indigenous Australians with non-small cell lung cancer or cervical cancer receive suboptimal treatment | NSCLC | Indigenous Australians | Retrospective cohort | Queensland Australia | 198 participants who were admitted to a Queensland Public Hospital. Indigenous with NSCLC (n=111) frequency-matched (by age, sex, ARIA, comparison group of non-Indigenous patients (n=87) | Clinical note review | Jan 1998 - Dec 2004 | Indigenous status checked in the medical record review |
